# Draft Genome Sequences of Legionella taurinensis Recovered from a Hot Water System in Austria, 2018

**DOI:** 10.1128/MRA.01478-18

**Published:** 2019-01-17

**Authors:** Ali Chakeri, Franz Allerberger, Michael Kundi, Anna Stöger, Sonja Rehak, Werner Ruppitsch, Daniela Schmid

**Affiliations:** a Institute of Medical Microbiology and Hygiene, Austrian Agency for Health and Food Safety, Vienna, Austria; b Center for Public Health, Medical University Vienna, Vienna, Austria; c Department of Biotechnology, University of Natural Resources and Life Sciences, Vienna, Austria; University of Maryland School of Medicine

## Abstract

Members of the genus *Legionella* are widespread in natural water sources. This is the first report on the draft genome sequences of Legionella taurinensis in Austria. The isolates were recovered from a hot water system associated with a case of Legionnaires’ disease caused by Legionella pneumophila in 2018.

## Abstract (precis)

Members of the genus *Legionella* are widespread in natural water sources. This is the first report on the draft genome sequences of Legionella taurinensis in Austria.

## ANNOUNCEMENT

The Gram-negative *Legionella* species are intracellular bacteria living in natural and man-made aquatic environments. The genus *Legionella* consists of 59 species and 70 serogroups (sg). Even though L. pneumophila sg1 causes more than 90% of reported Legionnaires’ disease cases (1, 2), infections with other *Legionella* species have been reported (3, 4).

Water samples from a hot water system of an apartment belonging to an Austrian patient infected with L. pneumophila were examined for *Legionella* as described (5). From each water sample, two portions were directly plated on buffered charcoal yeast extract (BCYE) and glycine vancomycin polymyxin cycloheximide (GVPC) agar (Oxoid, Hampshire, UK), another portion was membrane filtered (0.2-μm-pore polyamide filter; Millipore, Billerica, MA) with subsequent filter placement on GVPC agar, and a fourth portion was heat treated (50°C for 30 min in a water bath) and subsequently spread on a GVPC agar plate. The plates were incubated at 36°C in a humidified environment with 2.5% CO_2_ for 10 days and repeatedly inspected during the incubation. Colonies morphologically suspected to be *Legionella* were subcultured for species identification using a latex agglutination test (Oxoid, Hampshire, UK). The test did not reveal any of the species that were tested for, including L. pneumophila, L. longbeachae, L. bozemanae, L. dumoffii, L. gormanii, L. jordanis, L. micdadei, or L. anisa. For extraction of high-quality genomic DNA, five colonies from each water sample were subcultivated on GVPC and incubated for 48 h. Genomic DNA was extracted using the MagAttract high-molecular-weight (HMW) DNA kit (Qiagen, Hilden, Germany). A whole-genome sequencing library was prepared using an Illumina Nextera XT kit (Illumina, Inc., San Diego, CA), and paired-end sequencing (2 × 300 bp) was performed on a MiSeq system (Illumina, Inc.), generating 1,336,720 reads from 358,146,152 unassembled nucleotides and 1,182,150 reads from 311,991,780 unassembled nucleotides for isolate identification no. 4570-18-6 and 4567-18-4, respectively. Raw reads were *de novo* assembled using SPAdes version 3.11.1 (6) into a draft genome of 3,324,207 bp with 97 contigs, an *N*_50_ value of 164,180 bp, and a 47.9% GC content (4570-18-6) and a draft genome of 3,212,147 bp with 118 contigs, an *N*_50_ value of 213,110 bp, and a 45.9% GC content (4567-18-4). Contigs were filtered for a minimum coverage of 5× and a minimum length of 200 bp using SeqSphere+ (Ridom GmbH, Münster, Germany).

We used the NCBI Prokaryotic Genome Annotation Pipeline (7) for gene annotation and identified 3,026 total genes, 2,909 coding genes, 41 tRNAs, 4 noncoding RNAs (ncRNAs), and 62 pseudogenes (4570-18-6) and 3,110 total genes, 2,991 coding genes, 41 tRNAs, 4 ncRNAs, and 64 pseudogenes (4567-18-4).

Species identification was done via an NCBI 16S rRNA database BLAST search (8), and ribosomal multilocus sequencing typing (rMLST) was done via the PubMLST database (9). Both methods revealed 100% identity to L. taurinensis reference strain ATCC 700508. For phylogenetic analysis we generated an *ad hoc* core genome MLST scheme with 2,478 targets using the core genome MLST (cgMLST) target definer tool implemented in SeqSphere+ (10) and compared the Austrian strains with each other and with L. taurinensis reference strain ATCC 700508. We found the Austrian strains to differ from each other by 11 alleles and from the reference isolate ATCC 700508 by at least 53 alleles.

### Data availability.

The Legionella taurinensis whole-genome shotgun (WGS) projects have been deposited in DDBJ/ENA/GenBank under the accession no. QZWB00000000 (4570-18-6) and QZWC00000000 (4567-18-4). The versions described in this paper are the first versions, QZWB01000000 (4570-18-6) and QZWC01000000 (4567-18-4), and consist of sequences deposited under accession no. QZWB01000001 to QZWB01000118 (4570-18-6) and QZWC01000001 to QZWC01000097 (4567-18-4). SRA records are accessible under the accession no. PRJNA492387.

## References

[1] Fields BS, Benson RF, Besser RE 2002 *Legionella* and Legionnaires’ disease: 25 years of investigation. Clin Microbiol Rev 15:506–526. doi:10.1128/CMR.15.3.506-526.2002. 12097254 PMC118082

[2] Muder RR, Yu VL 2002 Infection due to *Legionella* species other than *L. pneumophila*. Clin Infect Dis 35:990–998. doi:10.1086/342884. 12355387

[3] Cunha BA, Cunha CB 2017 Legionnaire’s disease and its mimics: a clinical perspective. Infect Dis Clin North Am 31:95–109. doi:10.1016/j.idc.2016.10.008. 28159179

[4] Prochazka B, Indra A, Hasenberger P, Blaschitz M, Wagner L, Wewalka G, Sorschag S, Schmid D, Ruppitsch W 2016 Draft genome sequence of *Legionella jamestowniensis* isolated from a patient with chronic respiratory disease. Genome Announc 4:e01007-16. doi:10.1128/genomeA.01007-16. 27635013 PMC5026453

[5] European Guidelines Working Group. 2017 ECDC European Technical Guidelines for the Prevention, Control and Investigation of Infections Caused by Legionella Species. Accessed 9 July 2018 ECDC Digital Repository. https://ecdc.europa.eu/sites/portal/files/documents/Legionella%20GuidelinesFinal%20updated%20for%20ECDC%20corrections.pdf.

[6] Bankevich A, Nurk S, Antipov D, Gurevich AA, Dvorkin M, Kulikov AS, Lesin VM, Nikolenko SI, Pham S, Prjibelski AD, Pyshkin AV, Sirotkin AV, Vyahhi N, Tesler G, Alekseyev MA, Pevzner PA 2012 SPAdes: a new genome assembly algorithm and its applications to single-cell sequencing. J Comput Biol 19:455–477. doi:10.1089/cmb.2012.0021. 22506599 PMC3342519

[7] Tatusova A, DiCuccio M, Badretdin A, Chetvernin V, Nawrocki EP, Zaslavsky L, Lomsadze A, Pruitt K, Borodovsky M, Ostell J 2016 NCBI Prokaryotic Genome Annotation Pipeline. Nucleic Acids Res 44:6614–6624. doi:10.1093/nar/gkw569. 27342282 PMC5001611

[8] Zhang Z, Schwartz S, Wagner L, Miller W 2000 A greedy algorithm for aligning DNA sequences. J Comput Biol 7:203–214. doi:10.1089/10665270050081478. 10890397

[9] Jolley KA1, Bliss CM, Bennett JS, Bratcher HB, Brehony C, Colles FM, Wimalarathna H, Harrison OB, Sheppard SK, Cody AJ, Maiden MC 2012 Ribosomal multilocus sequence typing: universal characterization of bacteria from domain to strain. Microbiology 158:1005–1015. doi:10.1099/mic.0.055459-0. 22282518 PMC3492749

[10] Jünemann S, Sedlazeck FJ, Prior K, Albersmeier A, John U, Kalinowski J, Mellmann A, Goesmann A, von Haeseler A, Stoye J, Harmsen D 2013 Updating benchtop sequencing performance comparison. Nat Biotechnol 31:294–296. doi:10.1038/nbt.2522. 23563421

